# Dose frequency randomized controlled trial for Dynamic Temporal and Tactile Cueing (DTTC) treatment for childhood apraxia of speech: protocol paper

**DOI:** 10.1186/s12887-023-04066-2

**Published:** 2023-05-25

**Authors:** Jenya Iuzzini-Seigel, Julie Case, Maria I. Grigos, Shelley L. Velleman, Donna Thomas, Elizabeth Murray

**Affiliations:** 1 Department of Speech Pathology and Audiology, Marquette University, PO Box 1881, Harriet Barker Cramer Hall, Milwaukee, WI 53201 USA; 2 Speech-Language-Hearing Sciences, Hofstra University, Davison Hall 106B, 110, Hempstead, NY 11549 USA; 3 Communicative Sciences and Disorders, New York University, 665 Broadway, 9th floor, New York, NY 10012 USA; 4 University of Vermont, Pomeroy Hall, 489 Main St, Burlington, VT 05405 USA; 5 University of Sydney, Susan Wakil Health Building, Western Avenue, Camperdown, NSW 2006 Australia; 6 Remarkable Speech and Movement, 52 Anderson Avenue, Panania, NSW 2213 Australia

**Keywords:** Childhood apraxia of speech, Treatment, Motor speech, DTTC, Treatment schedule, Dose frequency, Randomized controlled trial

## Abstract

**Background:**

Childhood apraxia of speech (CAS) is a pediatric motor-based speech sound disorder that requires a specialized approach to intervention. The extant literature on the treatment of CAS commonly recommends intensive treatment using a motor-based approach, with some of the best evidence supporting the use of Dynamic Temporal and Tactile Cueing (DTTC). To date, a rigorous and systematic comparison of high and low dose frequency (i.e., frequency of therapy sessions) has not been undertaken for DTTC, resulting in a lack of evidence to guide decisions about the optimal treatment schedule for this intervention. The current study aims to fill this gap in knowledge by comparing treatment outcomes when dose frequency is varied.

**Methods:**

A randomized controlled trial will be conducted to examine the efficacy of low versus high dose frequency on DTTC treatment outcomes in children with CAS. A target of 60 children, 2;6–7;11 years of age, will be recruited to participate in this study. Treatment will be provided in the community setting by speech-language pathologists who have completed specialized training administering DTTC in a research reliable manner. True randomization with concealed allocation will be used to assign children to either the low or high dose frequency group. Treatment will be administered in 1-h sessions either 4 times per week over a 6-week period (high dose) or 2 times per week over a 12-week period (low dose). To measure treatment gains, probe data will be collected before treatment, during treatment, and 1 day, 1 week, 4 weeks, and 12 weeks post-treatment. Probe data will consist of customized treated words and a standard set of untreated words to assess generalization of treatment gains. The primary outcome variable will be whole word accuracy, encompassing segmental, phonotactic, and suprasegmental accuracy.

**Discussion:**

This will be the first randomized controlled trial to evaluate dose frequency for DTTC treatment in children with CAS.

**Trial registration:**

ClinicalTrials.gov identifier NCT05675306, January 6, 2023.

## Background

Childhood apraxia of speech (CAS) is a pediatric motor speech disorder that affects the planning and programming of speech movements [1, 2] and is estimated to occur in 1 per 1000 children [3]. Children with CAS typically have severe speech deficits that result in poor speech intelligibility, which will not resolve without appropriate targeted intervention (ASHA, [4]). There is a general consensus in the field that intense and frequent motor-based treatment is required to help remediate CAS [5–7]; however, empirical evidence specifying the optimal frequency of treatment sessions is lacking. While it seems intuitive to predict that more frequent sessions would result in greater treatment gains than less frequent sessions–even when cumulative dosage is kept constant–findings from previous research are conflicting [8]. The objective of the current work is to therefore examine the effect of dose frequency (i.e., frequency of therapy sessions) on treatment outcomes in Dynamic Temporal and Tactile Cueing (DTTC; [9]), one of the few evidence-based motor-based treatment approaches for CAS [6, 7].

### Dynamic Temporal and Tactile Cueing treatment (DTTC)

DTTC is a dynamic, motor-based treatment approach designed for children with severe CAS. This approach is based on Integral Stimulation [10–14]. In which the clinician instructs the child to “listen to me, watch me, and do what I do” and systematically supports the speech motor system to facilitate system-wide change in speech output [9]. In contrast to more traditional intervention approaches for speech sound disorders, the target of DTTC is speech movement gestures practiced in the context of real-word/phrase targets to optimize functional communication and be maximally motivating to the child. Within DTTC, the speech-language pathologist (SLP) engages the child in structured practice along a temporal hierarchy, where targets are first practiced at a slow rate and then progress to a regular rate as accuracy increases. Practice occurs across four levels: Simultaneous Production, Direct Imitation, Delayed Imitation and Spontaneous Production. At each level, the clinician dynamically varies the amount and type of supportive cueing provided, with the end goal being the child’s independent and accurate movement gestures within word- or phrase-level productions. Multisensory cueing (i.e., visual, tactile, gestural, verbal) is provided to support accurate speech production and cues are added/faded depending on the individual needs of the child. As the child becomes more independent in achieving accurate movement gestures, models and cues are faded to facilitate the child’s learning, retention, and generalization to untreated similar exemplars. Previous efficacy research on integral stimulation treatment, including DTTC, has generally used single-case experimental design studies and demonstrated moderate-to-large treatment and generalization effects for the majority of participants in the small samples studied [8, 10, 12, 13, 15–17]. Larger studies that control for or covary individual factors such as attention, severity, and resilience are needed to better understand what is driving varying responses to treatment for children in this heterogeneous population.

### Treatment intensity

Treatment intensity is a critical factor for optimizing speech motor learning [18]. There are a number of ways in which intensity can be operationalized [19] including the number of practice trials or teaching episodes per session (i.e., 'dose'; [10, 11]), number of total practice trials/teaching episodes over a period of time (i.e., ‘cumulative intervention intensity’; [20]), and frequency of treatment sessions (i.e., ‘dose frequency’; [21–23]). Investigations of treatment intensity in children with CAS reveal improved treatment outcomes when participants produce a higher number of trials per session [10] and when a greater number of practice trials are produced for a smaller stimulus set [11]. For instance, in a production frequency study of two children with CAS receiving integral stimulation treatment, Edeal and Gildersleeve-Neumann [10] reported better treatment outcomes for targets that were practiced 100 + times during each session compared to those practiced 30–40 times.

The extant literature on dose frequency in CAS is equivocal. Namasivayam, Pukonen [20] examined the impact of session frequency in 37 children with CAS within a play-based motor speech protocol therapy completed by community clinicians. Children received motor-based intervention once (low intensity) or twice per week (higher intensity), 60 min per session, for 10 weeks in community clinics. Findings revealed better treatment outcomes for the higher intensity, twice per week, condition in which children had received 20 sessions as compared to 10 sessions in the lower intensity group. Notably, no speech intelligibility gains were detected for either group, possibly suggesting that neither condition was sufficiently intense to promote these important gains. Leonhartsberger, Huber [8] also investigated the effect of session frequency on integral stimulation treatment outcomes in four German-speaking children with CAS when cumulative dosage was kept constant across conditions. Children were engaged in high intensity treatment (two 30-min treatment sessions daily for 2 weeks) followed by a period of low intensity intervention (one 60-min session each week for 10 weeks). Participants received a total of 10 h per treatment phase. Performance was compared between the high- and low-intensity conditions and revealed similar outcomes in each condition. Given that treatment order was not counter-balanced across children, it is unclear whether similar outcomes for both conditions could be attributed to a “jump-start” effect of the high-intensity condition. Thomas, McCabe [21] investigated outcomes of Rapid Syllable Transition (ReST) treatment provided two times per week over a 12-week period (*n* = 4). When compared to previous research that tested the efficacy of ReST administered 4 times per week over a 3-week period [22], analyses revealed similar initial treatment gains for both treatment intensities. However, children who received the more intense treatment schedule typically showed a rising profile even after treatment was completed whereas those who underwent the less frequent sessions showed no ongoing improvement once treatment ended.

Contrasting findings for treatment intensity have also been reported for children with non-CAS speech sound disorders [24]. High intensity intervention (3x/week over 8 weeks) yielded improved gains as compared to low intensity intervention (1x/week over 24 weeks) for a phonological intervention administered with the same cumulative dosage across groups [24]. In contrast to Thomas, McCabe [21], similar gains at maintenance were reported for both the high- and low- intensity groups. Taken together, these findings indicate a range for the effect of intensity on treatment outcomes within the CAS literature and other populations. Given that intensive treatment–specifically high frequency of clinical sessions per week–is widely recommended for children with CAS, it is critical to investigate how differing levels of intensity impact speech performance to provide evidence for clinical decision-making. Large scale, rigorous study of dose frequency in children with CAS is essential to determine whether high or low dose frequency is superior in effecting learning and generalization, or if schedule can be decided based on family and clinician preference and availability [25].

Previous work that examined treatment intensity in children with CAS, for various treatments, is limited by a variety of factors including small sample size [10, 11, 21], a lack of randomized control trials (RCTs) with true randomization (e.g., [20]), and under-specified protocols that make assessment, group assignment, and treatment difficult to replicate (cf. [26]). Consequently, it can be difficult to glean which treatment schedules will promote optimal learning and generalization outcomes for children with CAS.

### Current study

The proposed study aims to address these limitations by conducting an RCT to compare the effects of high and low dose frequency treatment schedules for a large sample of children with CAS undergoing DTTC speech treatment. To assess differences in treatment outcomes when dose frequency is varied, speech production accuracy will be tracked using whole word accuracy. The primary outcome measure will be whole word accuracy quantified using a multi-factor whole word accuracy measure, the Multilevel word Accuracy Composite Scale (MACS; [27]), which systematically accounts for segmental and prosodic accuracy, word shape maintenance, and smoothness and fluency of movement transitions. Secondary outcome measures will include phoneme accuracy (Percent Phonemes Correct; [28]), speech intelligibility (Intelligibility in Context Scale; [29]), and functional communication (Functional Outcomes on Communication Under Six; [30]). This RCT is expected to yield easily interpretable evidence to better understand the effect of dose frequency on treatment outcomes. We hypothesize that similar treatment and generalization gains will be detected for both the high and low dose frequency groups when cumulative dosage is constant (i.e., after 24 sessions have been completed). However, we predict that the high dose frequency group will demonstrate superior outcomes following 6 weeks of treatment when the high dose frequency group has completed 24 sessions and the low dose frequency group has completed 12 sessions. Based on our previous research [21, 22] and the extant literature, we also predict that the high dose frequency group will demonstrate superior maintenance of treatment and generalization gains compared to the low dose frequency group. Thus, while both conditions are predicted to yield positive treatment gains, we hypothesize the high dose frequency group to display these gains in a shorter timeframe and with greater maintenance and generalization as compared to the low dose frequency group.

## Methods and design

The proposed work is a multisite Phase III parallel-group RCT that aims to investigate the outcomes of DTTC in children with CAS (ages 2;6 to 7;11) and determine whether dose frequency impacts response to treatment. Participants will be randomly assigned to receive DTTC treatment at a low dose frequency (2x/week over 12 weeks) or high dose frequency (4x/week over 6 weeks). Group assignment will be performed using concealed randomization. All assessments and treatment will be provided by community SLPs throughout North America. All of the clinicians working on this study will undergo a rigorous application process, as well as extensive training including numerous applied activities. They will receive ongoing feedback from the investigators, who are SLPs with expertise in CAS, throughout the study to ensure high adherence to experimental protocols and fidelity to the operationalized assessment and treatment approaches.

### Ethical approval and consent to participate

This trial was registered on ClinicalTrials.gov, identifier NCT05675306, on January 6, 2023. Ethics approval for this study was obtained through the Marquette University Institutional Review Board (Protocol HR-4095). Informed consent to participate will be obtained from parents or caregivers on behalf of all participants. Assent will also be obtained from children who are 6 years of age and older.

### Targeted enrollment

We plan to enroll 60 children with CAS with potential to recruit an additional 6 children to account for a 10% rate attrition if needed. The power analysis for the treatment effect comparing the Lower Dose Frequency treatment group to the High Dose Frequency treatment group during the immediate-post testing period was done using a latent growth curve model [31, 32]. Specifically, the power analysis was conducted with respect to whole word accuracy, the primary outcome. Mplus [33] was used for power analysis with a sample size of 60 (30 per group). Mplus calculates power by simulating data given the design. In the simulation, the effect size is defined as change in the primary outcome between the low dose frequency group and the high dose frequency group divided by the square root of the model variance (a quantity similar to Cohen’s *d)*. At type one error of 5% (two tailed test) and a moderate treatment effect of 0.35, the power for estimating the treatment is 80%.

### Participants

This RCT is a multi-site study with up to 35 different community clinic treatment sites in the United States and Canada and 5 sites for data processing and analysis (Marquette University, Hofstra University, New York University, the University of Vermont, and the University of Sydney). Candidates for participation will be told about the study by their treating clinician using a script created by the research team. Alternatively, parents may respond to a flyer or postings on a website or social media account (e.g., Apraxia Kids). If the child’s parent/caregiver is interested in the study, they will complete the online screener to determine whether the child meets the minimum inclusionary criteria. The completed screeners will be reviewed by the research team. If a child meets screening criteria, the research team will contact the caregiver and describe the study and informed consent process in greater detail. If at that point the family would like to have their child participate, the research team will obtain a digital signature on the Informed Consent document using the HelloSign program; parental/caregiver informed consent to participate will be obtained for all participants. A digital signature will also be obtained on an assent document for children ages 6;0–7;11 (years;months). If additional support is needed to recruit our target of 60 participants, we will advertise through listservs, personal contacts, and flyers posted in public locations such as schools, pediatricians’ offices, and libraries. Participant enrollment will begin in January 2023.

Inclusionary criteria for child participants include (1) CAS diagnosis confirmed by research team as described below (e.g., [34–36]), (2) 2;6–7;11 years of age at treatment commencement; (3) English as the primary language; (4) no concomitant developmental disorders (including autism, global developmental delay, intellectual disability); (5) no diagnosis of severe or primary dysarthria as described below; (6) no palatal or structural orofacial anomalies as described below, (7) no uncorrected vision impairment (8) no hearing loss; (9) not receiving speech treatment elsewhere over the course of this study, although language, augmentative and alternative communication (AAC) treatment, or similar non-speech treatment, would be permitted (10) Receptive Language Index standard score greater than or equal to 70 on the Receptive-Expressive Emergent Language Test, 4^th^ edition (REEL-4; [37]) for children 2;6–2;11 years of age, the Clinical Evaluation of Language Fundamentals – Preschool 3^rd^ edition (CELF-P3; [38]) for children 3;0–5;11 years of age, or the Clinical Evaluation of Language Fundamentals – 5^th^ edition (CELF-5; [39]) for children 6;0–7;11 years of age, (11) Nonverbal Index standard score greater than or equal to 70 on the Developmental Assessment of Young Children-2^nd^ edition (DAYC-2; [40]) for children 2;6–5;11 years of age, the Reynolds Intellectual Assessment Scales– 2^nd^ edition, Remote (RIAS; [41]) for children 6;0–7;11 years old, and (12) evidence of communicative intent, attempts at verbal communication, focused attention to the clinician’s face, and demonstrated ability to imitate during the Dynamic Evaluation of Motor Speech Skill (DEMSS; [35]). All eligibility assessments will be administered by community speech-language pathologists and scored by the research team.

Participants will not be excluded from the proposed study on the basis of sex/gender or racial/ethnic group. Male and female children will be recruited, although we expect that males will enroll in higher numbers than females given that CAS occurs in 2–3 males per female [3].

### Assessment procedure

Consented participants will undergo a full diagnostic eligibility assessment and, if they qualify, will complete additional assessment tasks and be invited to undergo the treatment portion of the study. Parents/caregivers will complete a case history form about their child’s developmental, medical, and birth history, including brief information regarding their child’s siblings. If a child does not meet the inclusionary criteria after the eligibility assessment (e.g., due to deficits in language skills or hearing loss), they will be told that they do not meet criteria for participation in the treatment phase of the study. They will receive a $50 stipend for participating in the assessment.

The eligibility assessment protocol will include the Goldman Fristoe Test of Articulation-3rd edition (GFTA-3; [42]), a polysyllabic word test (Toddler Test of Polysyllables; [43]), an oral mechanism assessment, story retell of “Frog, where are you?” [44], and the DEMSS [35, 45]. If the child is unable to complete the story retell task, elicitation of a spontaneous speech sample will be attempted during play. Language will be assessed using the REEL-4 [37] for children under 3, the CELF-P3 [38] for children 3;0–5;11 years, or the CELF-5 [39] for children 6;0–7;11 years. The Intelligibility in Context Scale (ICS; [29], a 7-item parent survey, will be administered to assess communicative effectiveness. Resilience will be screened using the Child and Youth Resilience Measure (CYRM) – Revised ([46], Resilience Research Centre; [47]). Attention will be screened using the Conners Early Childhood Parent Survey [48] or the Conners-3 Parent Survey [49], depending on the child’s age. Nonverbal IQ will be assessed using the Developmental Assessment of Young Children (DAYC-2; [40]) in children 2;0–5;11 years of age or the Reynolds Intellectual Assessment Scales (RIAS; [41] in children 6;0–7;11 years of age. The Focus on the Outcomes of Communication in Children under Six (FOCUS-34; [30]) will be used to screen quality of life. Eligibility assessments will take place over the course of up to 3 1-h sessions, over 1–2 weeks.

#### Differential diagnosis of CAS

Differential diagnosis of CAS will be confirmed for each child by one member of the research team based on performance on the DEMSS, a dynamic speech assessment (e.g., [35]) and the presence of auditory-perceptual features consistent with CAS (e.g., [9, 34, 36]). Features associated with CAS and timing and coordination across speech subsystems will be observed on the following tasks: (1) dynamic speech assessment (i.e., DEMSS; [35]); (2) single word articulation testing (i.e., GFTA-3; [42]); (3) connected speech; (4) multisyllabic word productions (i.e., TPOT; [43]); (5) motor speech examination. An oral structural–functional examination will also be conducted to rule out any confounding craniofacial anomalies (e.g., submucosal cleft) or overt lower motor neuron dysarthria and to detect the presence of oral motor apraxia. The Profile of Childhood Apraxia of speech and Dysarthria (ProCAD; [34]) be used to identify articulatory and prosodic features that are discriminative of CAS and present in two or more speech contexts and to rule out the presence of features associated with dysarthria (motor execution issues) only. Performance across assessments will also be evaluated to rule out primary diagnosis of other speech sound disorders associated with an articulation or phonological impairment. Inter-rater reliability for differential diagnosis will be conducted on 10% of participants.

#### Group allocation

For participants who meet inclusionary criteria following the eligibility assessment, group allocation will be specified by the study statistician using a computer-generated random number list. The group assignments will be put into sequentially numbered, opaque, sealed envelopes with the specified group number indicated inside the envelope. This will help to conceal the randomization sequence until group assignments are made. The study statistician will generate confidential treatment assignments for participants 1 through 30 and participants will receive their group assignment in the order in which they are enrolled in the treatment phase. After 30 participants are enrolled, we will conduct preliminary group comparisons of participant demographics to determine the extent that the groups are balanced. If there are statistically significant differences between groups on potentially confounding demographic variables such as age, language ability, or speech severity, we will attempt to rebalance the groups for the subsequent 30 participants who are enrolled. Speech severity will be established through combined analysis of scores from dynamic motor speech skill assessment (DEMSS) and single word articulation testing (*GFTA-3*). In cases where a participant withdraws prior to the completion of the study, the next participant to enroll will be assigned to the same treatment condition to be the substitute. Participants and clinicians will be told which group each participant is assigned to by the research team after the informed consent document has been signed and eligibility has been determined by the eligibility assessments.

### Intervention delivery and dosage

DTTC treatment will be administered on an individualized basis by SLPs who were trained in DTTC by the research team, as described below. Treatment will take place in clinic rooms or at the child’s home in a quiet space. Treatment addressing functional communication, language goals, and the like will be allowed over this time period. Participants will receive 24 h of treatment, provided at no cost to the family. All sessions will be audio and video recorded for reliability and scoring purposes. We will report the number of participants initially recruited and tested, as well as those who did or did not meet inclusionary criteria in publications and presentations.

#### DTTC protocol

Participants in both conditions will receive DTTC treatment. In DTTC treatment, the target is accurate speech movement gestures in production of real words or phrases, rather than targeting accurate individual speech sounds as is common in traditional approaches for speech sound disorders [9]. Words and/or phrases are selected on an individual basis to target specific speech movement patterns and to be functional and motivating for each child (see Stimuli section below).

In DTTC, treatment targets are practiced along a temporal-based production hierarchy to provide varying degrees of support to facilitate speech accuracy [9]. Levels of the hierarchy include: (1) *Simultaneous Production –* the child and clinician produce targets at the same time*,* (2) *Direct Imitation –* the child produces the target immediately following the clinician’s model*,* (3) *Delayed Imitation –* the child produces the target following a brief delay after the clinician’s model*,* (4) *Spontaneous Production –* the child produces the target in response to questions or phrases. At lower levels of the hierarchy (i.e., *Simultaneous Production*, *Direct Imitation*), words are initially practiced at a reduced rate of speech to allow more time for the planning/programming of speech movements; practice gradually moves towards a regular rate as the child gains accuracy. Furthermore, prior to advancing along the temporal hierarchy, treatment targets are practiced while varying prosody (i.e., producing a target *happy/sad/mad/loud/soft/in a question*) to introduce practice variability and promote greater speech motor learning. Based on the individual needs of the child, the clinician provides multisensory cues (e.g., verbal, visual, tactile, gestural cues) to support production accuracy combined with frequent, specific feedback related to movement accuracy (i.e., knowledge of performance feedback). As the child becomes more accurate, clinician support and feedback are faded to only indicate accuracy of productions (i.e., knowledge of results feedback) and feedback is provided at a lower frequency.

During DTTC treatment sessions, a small set of treatment targets are practiced using a modified block schedule. A total of five words or phrases (i.e., treatment targets) will be practiced within any given treatment session with one treatment target produced individually at a time in either a small block (i.e., 15–24 words per block) or a large block (i.e., 25–40 words per block). The objective of each session is to achieve 2–3 blocks for each treatment target. There is a minimum of 50 productions per session required for each participant although we anticipate that there will be between 100–200 productions per session for each participant. In each session the clinicians will practice 5 treatment targets with 2–3 blocks per targeted word/phrase (total of 10–15 blocks across the session) with small blocks containing 15–25 productions and large blocks containing 25–40 productions. Clinicians will record each time a target was practiced and whether it was practiced in a small or large block.

At the beginning of each block, the clinician elicits a target in direct imitation and then uses clinical decision-making to determine the appropriate level of the temporal hierarchy depending on the degree of support needed for the targeted word/phrase (most support = *Simultaneous;* least support = *Spontaneous*). Practice may advance from *Simultaneous Production* to *Direct Imitation* once the child has accurately produced a treatment target 5–15 times at a regular rate of speech and the clinician has introduced varied prosody. After achieving 5–15 accurate productions at *Direct Imitation* with varied prosody, a target will be practiced at the level of *Delayed Imitation*. Practice will advance from *Delayed Imitation* to *Spontaneous Production* once a child has accurately produced a target 10–15 times with varied prosody. Once a word/phrase has been accurately produced at the level of *Spontaneous Production* 10/10 times across three consecutive sessions, that treatment target will be graduated out of active practice. The clinician will then refer to the Treatment Target bank to determine the next word or phrase item to include in treatment.

Dynamic assessment will be used to evaluate whether a word or phrase is ready for active treatment (e.g., stimulable with clinician support) and an appropriate substitute for graduated treatment targets. At the end of a treatment session during which a target has been graduated out of active practice, the clinician will select one word/phrase from the set of 20 potential treatment targets and briefly practice this target with the child. If the child responds to clinician cueing (e.g., verbal, tactile, visual, temporal cues), this word/phrase will be added to active treatment. If a child repeatedly attempts a word/phrase but productions remain equally inaccurate despite clinician cues, that target will not be introduced to treatment and the process will be repeated with a different word/phrase.

#### Clinicians

Community clinicians will be recruited to administer the assessment and treatment protocols. We anticipate having up to 35 different community clinicians across North America who practice in community clinics, schools, and private practice, primarily in the continental United States. Clinicians will undergo a rigorous application and training process including completion of 20 h of didactic and applied learning modules focused on assessment, treatment, research ethics, and study-specific protocols. Following the training and prior to collecting data, clinicians will achieve 90% fidelity in administration of the DEMSS and DTTC, achieve 90% accuracy or higher on an examination that assesses knowledge of the RCT’s experimental protocols and procedures, and achieve 90% reliability in making perceptual judgments of segmental and prosodic accuracy.

#### Stimuli

Stimuli will consist of individualized treatment targets that will be included in treatment sessions and a generalization corpus containing a common set of words that all children will produce. Treatment targets will consist of 20 words or phrases that may be targeted over the course of treatment. They will be selected by evaluating each child’s performance across assessment tasks, including their phonetic inventory, word shape inventory, lexical stress patterns, individual error profile with a specific focus on vowel errors, and responses to clinician cueing within dynamic assessment tasks (e.g., *DEMSS*). In addition, the child and/or their family will be consulted to ensure that potential treatment items are highly functional and motivating to the greatest extent possible. Targeted areas will include accuracy of movement gestures in a range of syllable and word shapes, vowel accuracy, coarticulatory contexts including a range of consonant and vowel transitions, and varied stress patterns.

The generalization corpus will be used to examine carryover of treatment gains. This set of words/phrases consists of 45 words/phrases that will not be included in treatment for any participant. These words/phrases have been divided into 15 low complexity targets, 15 medium complexity targets, and 15 high complexity targets. Target complexity was determined according to word structure, segmental features, and lexical/phrasal stress. Word structure of targets follows a hierarchy of increasing complexity determined by syllable/word shape (e.g., CV, VC, CVC, CVCV, VCVC, CCVC, CVCVC, etc.), syllable number (e.g., monosyllabic, bisyllabic, multisyllabic words and phrases), and presence of adjacent consonants (e.g., CCVC; CVC CVC). Segmental complexity accounts for age of acquisition (i.e., Early 8, Mid 8, Late 8; [50]), consonant features (i.e., place, manner, voicing), and vowel characteristics (i.e., vowel height/advancement; monophthong, diphthong). Complexity of movement transitions considers whether targets contain the same or different consonant–vowel sequences (i.e., same consonants/vowels, varied consonants/same vowels, same consonant/varied vowels, varied consonants/varied vowels) and the extent of place/manner/voicing changes across phoneme sequences. Suprasegmental complexity is based on whether lexical and phrasal stress follows either a trochaic or iambic pattern. See Table 1 for details regarding each level of complexity. Children with severe CAS will produce the 30 low and medium complexity targets. Those with moderate CAS will produce the 30 medium and high complexity targets. The child’s complexity level will be determined from their initial speech assessments using a checklist that clinicians will complete. The 15 medium complexity non-treatment words will be elicited from all participants.

**Table 1:** Target complexity framework

	**Word Structure**	**Segmental Complexity**	**Movement Sequences**	**Lexical Stress**
**Low Complexity**	CV VC CVC Reduplicated CVCV	**Consonants** Early consonants **Vowels** Simple vowels, diphtongs	Same consonant/vowel sequences	Trochaic
**Medium Complexity**	CVC CVCV VCVC	**Consonants** Early, mid, and some late consonants; Homorganic consonant clusters **Vowels** Simple vowels, diphthongs	Same and varied combinations of consonants (differing by place, manner, and/or voicing) and vowels	Mostly trochaic, some iambic
**High Complexity**	Multisyllabic words Phrases	**Consonants** Early, mid, and late consonants Homorganic & heterorganic consonant clusters **Vowels** Simple vowels, diphthongs	Varied combinations of consonants and vowels	Trochaic & iambic

#### Probe data

Treatment outcomes will be measured using probe data, which will be collected before, during, and following the intervention phase. The data collection schedule is presented in Table 2. Probe words consist of 50 items that include 20 individualized potential treatment targets and a 30-item generalization corpus. At each data collection session, one list of probe words will be presented in a randomized order with each word produced once. Probe data will be elicited in direct imitation (e.g., “Say, “apple") with no feedback or cues provided. The procedure for probe data collection is identical in baseline, treatment, and follow-up phases.

**Table 2:** Data collection schedule

	**Low dose frequency**			**High dose frequency**		
**Study week**	***Treatment***	***Assessment***	***Assessment evaluates***	***Treatment***	***Assessment***	***Assessment evaluates***
-3 to -2		P1 (× 1), ICS, FOCUS 34	Baseline accuracy, intelligibility and participation		P1 (× 1), ICS, FOCUS 34	Baseline accuracy, intelligibility and participation
-1		P2 (× 3)			P2 (× 3)	
1	Tx1 Tx2	P3 (× 1)	Baseline accuracy (pre-Tx1)	Tx1 Tx2 Tx3 Tx4	P3 (× 1)	
2	Tx3 Tx4	-		Tx5 Tx6 Tx7 Tx8	P4 (× 1)	Accuracy after 6 Tx sessions (pre-Tx7)
3	Tx5 Tx6	-		Tx9 Tx10 Tx11 Tx12	-	
4	Tx7 Tx8	P4 (× 1)	Accuracy after 6 Tx sessions (pre-Tx7)	Tx13 Tx14 Tx15 Tx16	P5 (× 1)	Accuracy after 12 Tx sessions (pre-Tx13)
5	Tx9 Tx10	-		Tx17 Tx18 Tx19 Tx20	P6 (× 1)	Accuracy after 18 Tx sessions (pre-Tx19)
6	Tx11 Tx12	-		Tx21 Tx22 Tx23 Tx24	P7 (× 2), ICS, FOCUS 34	Accuracy after 24 Tx sessions (1-day post-Tx24), intelligibility and participation
7	Tx13 Tx14	P5 (× 1)	Accuracy after 12 Tx sessions (pre-Tx7)		P8 (× 3)	1-week post-Tx accuracy
8	Tx15 Tx16	-				
9	Tx17 Tx18	-				
10	Tx19 Tx20	P6 (× 1)	Accuracy after 18 Tx sessions (pre-Tx19)		P9 (× 2), ICS, FOCUS 34	4-week post-Tx accuracy, intelligibility and participation
11	Tx21 Tx22	-				
12	Tx23 Tx24	P7 (× 2), ICS, FOCUS 34	Accuracy after 24 Tx sessions (1-day post-Tx24), intelligibility and participation			
13		P8 (× 3)	1-week post-Tx accuracy			
16		P9(× 2), ICS, FOCUS 34	4-week post-Tx accuracy, intelligibility and participation			
18					P10 (× 3)	12-week post-Tx accuracy
24		P10 (× 3)	12-week post-Tx accuracy			

*P* Probe session, *ICS* Intelligibility in Context Scale [29], *FOCUS 34* Focus on Communication in Under Six [30], *Tx* Treatment

##### Baseline phase

Probe data will be collected five times during the baseline phase. Baseline probe data collection will be spread across three separate sessions over the two-to-three-week period prior to the start of treatment. One set of probe data will be collected during the first baseline session (P1), three sets will be collected at the second baseline session (P2) with a 15-min break between administrations, and one set will be gathered immediately prior to the first treatment session (P3).

##### Treatment phase

Probe data will be collected four times across the treatment phase. One set of probe data will be collected in the first 10 min of the 7^th^, 13^th^, and 19^th^ treatment sessions (P4, P5, P6, respectively). Two sets of probe data (with a 15-min break between administrations) will be collected on the day following the final treatment session (P7).

##### Follow-up phase

Probe data will be collected at 1 week, 4 weeks, and 12 weeks following the last treatment session to measure treatment gains, maintenance, and generalization of gains. Three sets of probe data will be collected at 1-week post (P8), two sets of probe data will be collected at 4-weeks post (P9), and three sets will be collected at 12-weeks post (P10). There will be a 15-min break between administrations of probe data. The follow-up schedule was selected to facilitate comparison with other CAS treatment studies [21, 22, 51].

##### Additional assessments

Parents will complete the FOCUS-34 [30] and the ICS [29] one day following the last treatment session and four weeks post-treatment. The GFTA-3 [42] will be administered at one day post-treatment and four weeks post-treatment. Each of these sessions will take under 90 min, with breaks and game reinforcers given, as needed.

### Data management and storage

Eligibility and treatment data will be collected by community clinicians throughout the United States and Canada. All assessment and treatment sessions will be video recorded using an iPad 9 (64 GB, 10.2″ Display) and probe sessions will also be audio recorded. Once recordings are made, they will be transferred to SharePoint within 24 h to be processed, scored, transcribed, and stored by the research team.

### Statistical design

#### Outcome measures

The primary outcome measure is whole word accuracy which will be measured using the MACS scoring system [27]. The MACS was designed to specifically reflect areas of speech production most impacted in children with CAS through generating a composite score that reflects both segmental and suprasegmental accuracy at the whole-word level. The MACS rates production accuracy across the following categories: (1) segmental accuracy = accuracy of consonants and vowels; (2) word structure = maintenance of the targeted word shape; (3) prosody = accuracy of lexical stress in 2 + syllable words; (4) movement transitions = fluidity and smoothness of speech movements across sounds and syllables. Each of these categories is rated using a binary scale (0 = incorrect; 1 = correct). These ratings are then averaged to create the MACS composite score. The MACS score will be used to quantify production accuracy for all treatment targets and the generalization corpus produced within probe data collection. Whole word accuracy using the MACS will be measured across Baseline, Treatment, and Post-Treatment phases. Accuracy ratings will be compared between treated and untreated words to examine generalization effects [52].

Secondary outcome measures will include phoneme accuracy (Percent Phonemes Correct; [28]), speech intelligibility (ICS; [29]), and functional communication (FOCUS-34; [30]). Measures of speech intelligibility and functional communication will be completed by caregivers pre- and post-treatment.

#### Data preparation

Ratings of whole word accuracy and phoneme accuracy will be completed by trained research assistants based on digital recordings obtained using a Zoom H1n 2-channel Handy Recorder (see Recording and Equipment below). A guided tutorial will be completed for training in the use of MACS ratings to measure speech accuracy in children with speech impairment [27]. Raters will be expected to achieve at least 90% agreement with laboratory ratings completed by two of the primary investigators who developed this measure and have expertise in rating speech produced by children with CAS (JC, MG). Probe word productions will also be transcribed in Phon [53]. Prior to transcribing data for the study, research assistants will undergo a rigorous training protocol in the transcription of disordered speech and will be required to demonstrate ≥ 90% agreement with consensus ratings completed by expert raters. Research assistants will be blinded to group and time point when transcribing participant probes through the use of deidentified, coded file names that lack any information that can be used to glean the child’s group, the session date, or other pertinent information. They will have access to a recording of a family member of the child with the same dialect producing the probe items as an anchor if they need help determining an error versus dialectal difference. Within Phon, research assistants will (a) transcribe the phonemes, noting any non-allophonic variation (e.g., lengthening, nasality, distortions); (b) record primary and secondary stress within each item, (c) note instances of excess syllable segregation within and between words, (d) enter the MACS score for each item. Inter- and intra-rater reliability will be calculated on a randomly selected 20% of probes. To confirm that raters are maintaining reliability with one another, inter-rater reliability will be assessed once each rater has completed 10 probe sessions. The intraclass correlation coefficient (ICC) will be used to measure reliability between raters. If raters have not maintained “good” inter-rater reliability (i.e., ICC values between 0.75–0.90; [54]), consensus ratings will be performed on sessions that did not achieve adequate reliability.

### Statistical analysis plan

A Quasi-Poisson regression model will be used to calculate the effect size for the primary outcome measure of whole word accuracy (i.e., the MACS score). This model will be used to account for changes in the relationship between the mean and variance across various stages of treatment. A standard effect size assumes independence between the mean and variance. However, past work [55] has demonstrated that these values are not independent of one another across the experimental period (e.g., at post-treatment, higher accuracy often occurs in combination with more stable performance as compared to baseline where low accuracy occurs in combination with more variable productions). The quasi-Poisson model accounts for these changes in the relationship between the mean and variance to more accurately calculate effect sizes. Gains in whole-word accuracy will be calculated from Baseline to 1 day and 1 week post to capture treatment effects and from Baseline to 4 weeks post and 12 weeks post, respectively, to examine maintenance of treatment gains.

Given that multiple data points will be collected over time, data will also be analyzed using longitudinal growth curve modeling within the framework of Structural Equation Modeling [56, 57]. Growth curve modeling was chosen for its capacity of modeling within-participant change over time, as well as between-participant differences. It estimates the mean intercept and mean slope for the sample. Similar to the basic regression model, these effects are assumed fixed for all individuals in the sample. It also takes into account within-participant correlation among the repeated measurements by incorporating random components (i.e., individual variations around the mean intercept and around the mean slope).

Growth curve modeling considers change over time as an underlying latent process. In analyzing this process, a trajectory of change over time is established for each individual in a sample, and therefore, characteristics of the trajectory (e.g., slope) may vary across individuals and are treated as latent variables. These latent variables describe parameters of change and may be treated as independent, dependent, control, or mediating variables and be compared across groups. Specifically, the growth curve model will allow us to (a) compare the treatment effect between Low Dose Frequency treatment and High Dose Frequency treatment groups during the treatment stage; (b) compare groups on maintenance effects during the post-treatment stage; (c) explain variations in change by incorporating various predictors. The growth curve model will also allow us to model flexible patterns of change in time such as quadratic and piecewise forms. Analyses will be conducted using Mplus software 5.0 [33].

Throughout our modeling process, we will carefully consider potential confounders that should be added to the model as covariates, such as severity of impairment, number of blocks practiced per session, age, and sex of the children. In the presence of attrition, an intention to treat analysis will be performed and compared with the analysis done on the “per protocol” population whenever possible. Baseline characteristics of the completed and withdrawn participants will be compared to prevent selection bias in the conclusions. In addition, internal validity (i.e., how well the randomization worked to create similar study groups) will be checked by comparing the groups on relevant measures using analyses of variance.

### Data cleaning and preparation

Data will be securely stored and transferred in SharePoint folders. Trained research assistants will enter data into spreadsheets using participant identifiers. All data will be reviewed for valid values/data entry errors, outliers, and the extent and patterns of missing data. Consistency and logic checks that constitute standard review/cleaning procedures will be applied. The distributions of these measures across sessions will be summarized. Descriptive statistics will be used to describe measures at different periods in the study. Growth curve models allow us to quantify change from pre- to post-treatment and post-treatment to maintenance by interacting the treatment effect with time. Effect sizes for treatment will be compared between Low Dose Frequency treatment and High Dose Frequency treatment. The growth curve of each individual participant will be fit through random effect components, which control intra-participant correlation introduced by repeated measures. Covariates will also be included to control for any covariate imbalance between different treatment groups.

#### Missing values

Missing data will be documented, as well as reasons as to why the data is missing (e.g., experimenter error, missed session, poor audio quality) where available. Upon completion of data collection, we will evaluate whether missing data is a random occurrence or due to a specific reason. If a recurring reason for missing data is determined, this variable will be coded and included within statistical modeling as a co-variate.

### Treatment fidelity

Fidelity to the treatment protocol will be assessed in 20% of treatment sessions for each child according to the following schedule: (1) 30 min of each of the first 3 treatment sessions, (2) an additional 3.3 h of treatment sessions randomly selected from the remainder of the intervention phase. A member of the research team will review these sessions using a DTTC fidelity checklist to determine adherence to the experimental treatment protocol. If clinicians fall below 90% fidelity during a treatment session, additional fidelity sessions will be scheduled with individualized feedback provided by one of the PIs. Once the clinician has achieved 90% fidelity, the planned fidelity schedule will be resumed.

### Recording and equipment

Each clinical site will record audio and video for all assessment and treatment sessions using an iPad (9^th^ generation) with a cardioid directional externally mounted microphone (Samson Satellite iOS/USB Broadcast Microphone). The iPad will be mounted on a UBeesize 50″ Extendable Lightweight Aluminum tripod stand located between 18 inches and 2 feet from the child. In addition, each clinical site will use a Zoom H1n 2-channel Handy Recorder audio recorder to capture probe data collection and to serve as a back-up recording for eligibility assessments. These recordings will be collected at a 96 kHz sampling rate with 24-bit encoding and saved to a Samsung – PRO 32 GB microSDXC UHS-I Memory Card.

## Discussion

### Potential significance

When the proposed study is complete, we will have measured treatment effects and generalization of DTTC in 60 children randomly assigned to groups with the same cumulative dosage that differ in dose frequency (i.e., low dose = treatment 2x/week for 12 weeks vs. high dose = treatment 4x/week for 6 weeks). Based on the extant literature [8, 22], we posit that both groups will demonstrate comparable improvements following the full dose (24 h) of treatment, but that between-group differences will be detected at other measurement points. We predict a group-by-time interaction, wherein the high dose group will demonstrate a significant advantage after 6 weeks of treatment as they will have completed 24 treatment hours compared to the low dose group, which will have completed 12 h of DTTC at that point. We also anticipate a group-by-time interaction for maintenance effects in which the high dose group will evidence superior maintenance of treatment and generalization gains compared to the low dose frequency group at the 4-week and 12-week post treatment follow-up sessions. If these hypotheses are correct, this study will provide evidence that DTTC treatment provided at a higher dose frequency yields faster and continued gains even after treatment is discontinued. More efficient service delivery will help children to progress through treatment in fewer years to develop functional speech. This may help reduce the risk of social-emotional challenges including anxiety and depression (e.g., [58, 59]). It may also result in the provision of better early intervention services and reduce long-term academic and vocational disparities for people with CAS [60, 61]. Alternatively, if treatment effects are shown but group differences are not detected, this will suggest that treatment schedule can be determined based upon available resources and family/clinician preference.

### Potential limitations

As noted above, assessments and treatment in the proposed study will be conducted by community speech-language pathologists. Although engaging community clinicians in this study will help to increase the application, external validity, and translation of this research, it may result in potential limitations as well. To start, DTTC is considered a dynamic and individualized treatment that relies on the clinician’s ability to monitor and cue the child to promote speech accuracy. The clinical experience of our community clinicians will vary which could introduce clinician effects. However, the randomization of participants to groups should help to minimize any effects of the clinician or clinical environment. In addition, to help address the varied expertise of the clinician partners working in this study, all clinicians will be required to undergo 20 h of training focused on assessment and treatment protocols and study procedures. Fidelity measures will also ensure that all clinicians, regardless of their prior experience, will adhere to the study protocols. Additional training and guidance will be given if needed. Clinicians will also meet in a group with the research team twice per month for ongoing training and problem-solving as needed. There is also the possibility that children may be challenged with the intensive treatment schedule and 60-min sessions where they are engaged in structured practice. As DTTC is focused on conscious practice of speech production, children may become frustrated and fatigued during sessions. As part of the clinical training and group meetings, strategies have been presented to support clinicians in their ability to maintain engagement, build trust and rapport, and practice at optimal challenge levels for each individual child. Group meetings will also offer clinicians the opportunity to review clinical cases and approaches for supporting children across the treatment phase.

Clinical research conducted in a lab often has the benefit of employing research assistants to help manage audio-visual recording for probes and treatment sessions, which helps to ensure high-quality recordings and to minimize data loss. By contrast, the community clinicians employed in this study will have to manage clinical care along with recordings and other logistics, which may result in occasional data loss if a video recording is not started appropriately or if there is a technical difficulty. To prevent missing data, all probes will also be audio recorded with a separate device as backup in case the video recording fails. Other safeguards will also be in place to ensure that clinicians provide assessments and treatment in a standardized way while also managing the many other requirements of research data collection. Examples of these safeguards include emails or text messages sent to clinicians prior to probe sessions as a reminder of tasks that need to be completed, daily session checklists indicating all activities to be completed during each session, and a study manual that provides detailed, operationalized protocols and procedures.

Difficulty with recruitment is also a potential challenge. While CAS is a relatively rare disorder occurring in only 1/1000 children [3], by including over 20 community clinicians across North America, we should be able to recruit the 60 participants required for the proposed study. We anticipate that some families may prefer to come 4 times per week while others may view that as too much of a burden and would prefer to come only twice a week. Given that this is an RCT, family preference will not be a factor in group assignment. To help support families who may be concerned about transportation and/or childcare of their other children while they are bringing a participant to treatment, a stipend of $40 will be provided for each session the child attends.

This study will compare two different treatment schedules and, consequently, it is imperative that children attend according to the prescribed schedule. While missing sessions due to illness such as COVID-19 cannot be avoided, it is possible to schedule study participation around other commitments such as vacations and holidays. To avoid preventable absences from treatment and follow-up sessions and ensure adherence to the study protocol, each clinician will work through the entirety of the study schedule with families prior to conducting baseline testing. Families will sign off in agreement to all dates from baseline through the 12-week follow-up before these activities begin.

## Data Availability

Materials referenced in this protocol are available in an Open Science Framework.

## Abbreviations

DTTC: Dynamic Temporal and Tactile Cueing
CAS: Childhood apraxia of speech
ASHA: American Speech-Language-Hearing Association
SLP: Speech-language pathologist
ReST: Rapid Syllable Transition
RCT: Randomized control trial
MACS: Multilevel word Accuracy Composite Scale
PPC: Percent Phonemes Correct
ICS: Intelligibility in Context Scale
FOCUS: Functional Outcomes on Communication Under Six
REEL-4: Receptive-Expressive Emergent Language Test, 4^th^ Edition
DAYC-2: Developmental Assessment of Young Children – 2^nd^ Edition
RIAS-2^nd^ Edition, Remote: Reynolds Intellectual Assessment Scales-2^nd^ Edition, Remote
DEMSS: Dynamic Evaluation of Motor Speech Skill
GFTA: Goldman Fristoe Test of Articulation
CELF-P3: Clinical Evaluation of Language Fundamentals-Preschool, 3^rd^ Edition
CELF-5: Clinical Evaluation of Language Fundamentals-5^th^ Edition
CYRM: Child and Youth Resilience Measure
T-POT: Toddler Polysyllable Test
ProCAD: Profile of Childhood Apraxia of speech and Dysarthria
ML: Maximum Likelihood
WLSMV: Weighted Least Squares Means and Variance adjusted estimator
COVID: Corona virus disease

## References

[1] Grigos MI, Moss A, Lu Y (2015). Oral articulatory control in childhood apraxia of speech. J Speech Lang Hear Res.

[2] Terband H, Maassen B, van Lieshout P, Nijland L (2011). Stability and composition of functional synergies for speech movements in children with developmental speech disorders. J Commun Disord.

[3] Shriberg LD, Kwiatkowski J, Mabie HL (2019). Estimates of the prevalence of motor speech disorders in children with idiopathic speech delay. Clin Linguist Phon.

[4] American Speech-Language-Hearing Association. Childhood apraxia of speech [Technical Report]. 2007. Available from www.asha.org/policy/.

[5] Morgan AT, Murray E, Liégeois FJ (2018). Interventions for childhood apraxia of speech. Cochrane Database Syst Rev.

[6] Murray E, McCabe P, Ballard KJ (2014). A systematic review of treatment outcomes for children with childhood apraxia of speech. Am J Speech Lang Pathol.

[7] Maas E, Gildersleeve-Neumann C, Jakielski K, Stoeckel R (2014). Motor-based intervention protocols in treatment of childhood apraxia of speech (CAS). Curr Dev Disord Rep.

[8] Leonhartsberger S, Huber E, Brandstötter G, Stoeckel R, Baas B, Weber C (2022). Efficacy of treatment intensity in German-speaking children with childhood apraxia of speech. Child Lang Teach Ther.

[9] Strand EA (2020). Dynamic Temporal and Tactile Cueing (DTTC): A Treatment Strategy for Childhood Apraxia of Speech. Am J Speech Lang Pathol.

[10] Edeal DM, Gildersleeve-Neumann CE (2011). The importance of production frequency in therapy for childhood apraxia of speech. Am J Speech Lang Pathol.

[11] Maas E, Gildersleeve-Neumann C, Jakielski K, Kovacs N, Stoeckel R, Vradelis H (2019). Bang for Your Buck: A Single-Case Experimental Design Study of Practice Amount and Distribution in Treatment for Childhood Apraxia of Speech. J Speech Lang Hear Res.

[12] Maas E, Farinella KA (2012). Random versus blocked practice in treatment for childhood apraxia of speech. J Speech Lang Hear Res.

[13] Strand E, Debertine P (2000). The efficacy of integral stimulation intervention with developmental apraxia of speech. J Med Speech-Lang Pathol.

[14] Strand EA, Skinder A, Caruso A, Strand E (1999). Treatment of developmental apraxia of speech: Integral stimulation methods. Clinical management of motor speech disorders in children.

[15] Strand EA, Stoeckel R, Baas B (2006). Treatment of severe childhood apraxia of speech: A treatment efficacy study. J Med Speech-Lang Pathol.

[16] Maas E, Butalla CE, Farinella KA (2012). Feedback frequency in treatment for childhood apraxia of speech. Am J Speech Lang Pathol.

[17] Baas BS, Strand EA, Elmer LM, Barbaresi WJ (2008). Treatment of severe childhood apraxia of speech in a 12-year-old male with CHARGE association. J Med Speech-Lang Pathol.

[18] Maas E, Robin DA, Hula SNA, Freedman SE, Wulf G, Ballard KJ (2008). Principles of motor learning in treatment of motor speech disorders. Am J Speech Lang Pathol.

[19] Warren SF, Fey ME, Yoder PJ (2007). Differential treatment intensity research: A missing link to creating optimally effective communication interventions. Ment Retard Dev Disabil Res Rev.

[20] Namasivayam AK, Pukonen M, Goshulak D, Hard J, Rudzicz F, Rietveld T (2015). Treatment intensity and childhood apraxia of speech. Int J Lang Commun Disord.

[21] Thomas DC, McCabe P, Ballard KJ (2014). Rapid syllable transitions (ReST) treatment for childhood apraxia of speech: The effect of lower dose-frequency. J Commun Disord.

[22] Murray E, McCabe P, Ballard KJ (2015). A randomized controlled trial for children with childhood apraxia of speech comparing rapid syllable transition treatment and the Nuffield Dyspraxia Programme. J Speech Lang Hear Res.

[23] Namasivayam AK, Pukonen M, Goshulak D, Vickie YY, Kadis DS, Kroll R (2013). Relationship between speech motor control and speech intelligibility in children with speech sound disorders. J Commun Disord.

[24] Allen MM (2013). Intervention efficacy and intensity for children with speech sound disorder.

[25] Dollaghan CA. The handbook for evidence-based practice in communication disorders: Paul H Brookes Publishing; 2007.

[26] Murray E, McCabe P, Ballard KJ (2012). A comparison of two treatments for childhood apraxia of speech: Methods and treatment protocol for a parallel group randomised control trial. BMC Pediatr.

[27] Case J, Wang E, Grigos MI. The multilevel word accuracy composite scale: a novel approach to rating speech errors in Childhood Apraxia of Speech (CAS). Am J Speech Lang Pathol. 2023;1–18. 10.1044/2023_AJSLP-22-00166 PMC10561970 37195724

[28] Shriberg LD, Austin D, Lewis BA, McSweeny JL, Wilson DL (1997). The percentage of consonants correct (PCC): Metric extensions and reliability data. J Speech Lang Hear Res.

[29] McLeod S, Harrison LJ, McCormack J (2012). The intelligibility in context scale: Validity and reliability of a subjective rating measure.

[30] Thomas-Stonell N, Washington K, Oddson B, Robertson B, Rosenbaum P (2013). Measuring communicative participation using the FOCUS©: Focus on the Outcomes of Communication Under Six. Child Care Health Dev.

[31] DeLucia C, Pitts SC (2006). Applications of individual growth curve modeling for pediatric psychology research. J Pediatr Psychol.

[32] Price M, Anderson PL (2011). Latent growth curve analysis of fear during a speech task before and after treatment for social phobia. Behav Res Ther.

[33] Muthén B, Asparouhov T (2002). Latent variable analysis with categorical outcomes: Multiple-group and growth modeling in Mplus. Mplus web notes.

[34] Iuzzini-Seigel J, Allison KM, Stoeckel R (2022). A tool for differential diagnosis of childhood apraxia of speech and dysarthria in children: a tutorial. Lang Speech Hear Serv Sch.

[35] Strand EA, McCauley R (2018). Dynamic Evaluation of Motor Speech Skill Manual.

[36] Shriberg LD, Strand EA, Fourakis M, Jakielski KJ, Hall SD, Karlsson HB, et al. A diagnostic marker to discriminate childhood apraxia of speech from speech delay: I. Development and description of the pause marker. J Speech Lang Hear Res. 2017;60(4):S1096–117. 10.1044/2016_JSLHR-S-15-0296 PMC5548086 28384779

[37] Brown V, Bzoch K, League R. Receptive–Expressive Emergent Language Test: Pro-Ed; 2020.

[38] Wiig EH, Secord W, Semel EM (2020). Clinical Evaluation of Language Fundamentals - Preschool.

[39] Wiig EH, Semel EM, Secord W (2013). Clinical Evaluation of Language Fundamentals.

[40] Voress JK, Maddox T. Developmental Assessment of Young Children: DAYC. 2nd edition ed. Austin, TX: Pro-ed 2013.

[41] Reynolds C, Kamphaus R (2015). Reynolds Intellectual Assessment Scales (2. Baskı).

[42] Goldman R, Fristoe M. Goldman Fristoe Test of Articulation. 3rd ed. Bloomington, IN: Pearson; 2015.

[43] Baker E (2017). Toddler Test of Polysyllables (T-POT for GAE): University of Sydney.

[44] Mayer M (1969). Frog, where are you? New York.

[45] Strand EA, McCauley RJ, Weigand SD, Stoeckel RE, Baas BS (2013). A motor speech assessment for children with severe speech disorders: Reliability and validity evidence. J Speech Lang Hear Res.

[46] Jefferies P, McGarrigle L, Ungar M (2019). The CYRM-R: A Rasch-validated revision of the child and youth resilience measure. J Evid Based Soc Work.

[47] Centre RR (2018). CYRM and ARM User Manual: Resilience Research Centre.

[48] Conners CK (2009). Conners Early Childhood™.

[49] Conners CK (2008). Conners 3: Pearson.

[50] Crowe K, McLeod S. Children’s english consonant acquisition in the United States: a review. Am J Speech Lang Pathol. 2020;29(4):2155–69. 10.1044/2020_AJSLP-19-00168 33181047

[51] Thomas DC, McCabe P, Ballard KJ, Lincoln M (2016). Telehealth delivery of Rapid Syllable Transitions (ReST) treatment for childhood apraxia of speech. Int J Lang Commun Disord.

[52] Baker E, Masso S, Huynh K, Sugden E (2022). Optimizing outcomes for children with phonological impairment: a systematic search and review of outcome and experience measures reported in intervention research. Lang Speech Hear Serv Sch.

[53] Hedlund G, Rose Y. Phon 3.1. [Computer Software].

[54] Koo TK, Li MY (2016). A guideline of selecting and reporting intraclass correlation coefficients for reliability research. J Chiropr Med.

[55] Grigos MI, Case J, Lu Y. Dynamic Temporal and Tactile Cueing (DTTC) in young children with childhood apraxia of speech: A single case experimental design. Am J Speech Lang Pathol. Under review. 10.1044/2024_JSLHR-23-00415 PMC11005957 38512002

[56] Duncan ES, Schmah T, Small SL (2016). Performance variability as a predictor of response to aphasia treatment. Neurorehabil Neural Repair.

[57] Willett JB, Sayer AG (1994). Using covariance structure analysis to detect correlates and predictors of individual change over time. Psychol Bull.

[58] Cassar C, McCabe P, Cumming S. “I still have issues with pronunciation of words”: a mixed methods investigation of the psychosocial and speech effects of childhood apraxia of speech in adults. Int J Speech Lang Pathol. 2022;25(2):193–205. 10.1080/17549507.2021.2018496 35034534

[59] Carrigg B, Parry L, Baker E, Shriberg LD, Ballard KJ (2016). Cognitive, linguistic, and motor abilities in a multigenerational family with childhood apraxia of speech. Arch Clin Neuropsychol.

[60] Lewis BA, Freebairn LA, Hansen AJ, Iyengar SK, Taylor HG (2004). School-age follow-up of children with childhood apraxia of speech. Lang Speech Hear Serv Sch.

[61] Miller GJ, Lewis B, Benchek P, Freebairn L, Tag J, Budge K (2019). Reading outcomes for individuals with histories of suspected childhood apraxia of speech. Am J Speech Lang Pathol.

